# Efficient Targeting of Head and Neck Squamous Cell Carcinoma by Systemic Administration of a Dual uPA and MMP-Activated Engineered Anthrax Toxin

**DOI:** 10.1371/journal.pone.0020532

**Published:** 2011-05-31

**Authors:** Jeffrey M. Schafer, Diane E. Peters, Thomas Morley, Shihui Liu, Alfredo A. Molinolo, Stephen H. Leppla, Thomas H. Bugge

**Affiliations:** 1 Oral and Pharyngeal Cancer Branch, National Institute of Dental and Craniofacial Research, National Institutes of Health, Bethesda, Maryland, United States of America; 2 Program of Pharmacology and Experimental Therapeutics, Tufts University School of Medicine, Boston, Massachusetts, United States of America; 3 Bacterial Toxins and Therapeutics Section, Laboratory of Bacterial Diseases, National Institute of Allergy and Infectious Diseases, National Institutes of Health, Bethesda, Maryland, United States of America; Ludwig-Maximilians University, Germany

## Abstract

Head and neck squamous cell carcinoma (HNSCC) is the sixth most common cancer worldwide. Although considerable progress has been made in elucidating the etiology of the disease, the prognosis for individuals diagnosed with HNSCC remains poor, underscoring the need for development of additional treatment modalities. HNSCC is characterized by the upregulation of a large number of proteolytic enzymes, including urokinase plasminogen activator (uPA) and an assortment of matrix metalloproteinases (MMPs) that may be expressed by tumor cells, by tumor-supporting stromal cells or by both. Here we explored the use of an intercomplementing anthrax toxin that requires combined cell surface uPA and MMP activities for cellular intoxication and specifically targets the ERK/MAPK pathway for the treatment of HNSCC. We found that this toxin displayed strong systemic anti-tumor activity towards a variety of xenografted human HNSCC cell lines by inducing apoptotic and necrotic tumor cell death, and by impairing tumor cell proliferation and angiogenesis. Interestingly, the human HNSCC cell lines were insensitive to the intercomplementing toxin when cultured *ex vivo*, suggesting that either the toxin targets the tumor-supporting stromal cell compartment or that the tumor cell requirement for ERK/MAPK signaling differs *in vivo* and *ex vivo*. This intercomplementing toxin warrants further investigation as an anti-HNSCC agent.

## Introduction

With more than 500,000 new cases diagnosed each year, head and neck squamous cell carcinoma (HNSCC) represents the sixth most common cancer worldwide [1]. Although the risk factors and the molecular pathways that underlie HNSCC development are by now well-known, the five-year survival rate after diagnosis is low and has remained unchanged for many decades [2]–[6].

The overexpression, prognostic significance, and causal involvement of extracellular/pericellular proteases in the progression of HNSCC have been extensively studied (reviewed in [7]–[10]). The multiple proteases that are expressed at very high levels by either tumor cells, stromal cells or both include urokinase plasminogen activator (uPA), tissue plasminogen activator, matrix metalloproteinase (MMP)-1, -2, -3, -7, -9, -10, -11, -13, and -14, cathepsins B, D, H, and L, kallikreins 5, 7, 8, and 10, and matriptase [11]–[32].

Anthrax toxin is a three-component toxin secreted by *Bacillus anthracis* that consists of protective antigen (PA, 83 kDa), lethal factor (LF, 90 kDa), and edema factor (EF, 90 kDa) [33]. To intoxicate cells, PA binds to either of two widely-expressed cell surface receptors, tumor endothelial marker 8 (TEM8, also “ANTXR1”) or capillary morphogenesis gene 2 product (CMG2, also “ANTXR2”) [34]–[37], and subsequently is cleaved at the sequence ^164^RKKR^167^ by cell-surface furin or furin-like proteases, which are ubiquitously expressed by cells [38], [39]. This cleavage is essential for all subsequent steps of intoxication. The newly generated C-terminal 63-kDa fragment of PA remains bound to its cell surface receptor and forms a heptamer that binds and translocates up to three molecules of LF or EF into the cytosol to induce their cytotoxic effects. EF is a potent adenylate cyclase that intoxicates cells by raising cAMP levels, whereas LF is a metalloproteinase that cleaves and inactivates mitogen-activated protein kinase kinases (MEKs), thereby blocking the extracellular signal-regulated kinase (ERK)/mitogen activated protein kinase (MAPK) pathway [40]–[45].

Strategies to therapeutically exploit the signature overexpression of proteolytic enzymes in cancer have mostly focused on inhibiting their enzymatic activity so as to blunt invasive and metastatic processes [46]–[48]. More recently, however, strategies have been devised to generate tumor cytotoxic pro-drugs that are activated by specific tumor-expressed proteases [49]–[51]. In this regard, we previously generated a reengineered intercomplementing anthrax toxin that requires the combined activities of both cell surface uPA and MMP for cytotoxicity, and showed that this toxin has a greatly diminished off-target cytotoxicity when compared to native anthrax toxin [52]. The intercomplementing toxin consists of PA-U2-R200A, which is a reengineered PA that is activated by uPA and PA-L1-I210A, which is a reengineered PA that is activated by MMPs. PA-U2-R200A and PA-L1-I210A also each harbor additional, but different, mutations in the LF/EF binding site that make heptamers composed of PA-U2-R200A alone or of PA-L1-I210A alone unable to bind EF or LF and support their translocation to the cytoplasm. However, heptamers that are composed of a mixture of PA-U2-R200A and PA-L1-I210A can form functional EF/LF binding sites in by intermolecular complementation.

Because of the consistent expression of both uPA and several MMPs by human HNSCC, here we explored the potential use of this toxin as a novel targeted treatment for this disease. Indeed, we found that systemic administration of the intercomplementing toxin caused regression of several xenografted human HNSCC. Interestingly, the *in vivo* efficacy of the toxin was independent of the sensitivity of the cultured tumor cells to the toxin, suggesting that the intercomplementing toxin may inhibit tumor growth by targeting both the tumor cell compartment and the stromal cell compartment.

## Methods

### Ethics statement

All animal work was performed in accordance with protocols approved by the National Institute of Dental and Craniofacial Research Animal Care and Use Committee (Animal Study Proposal Numbers: 09-523 and 10-585).

### Protein purification

Recombinant PA, PA-U2-R200A, PA-L1-I210A, LF, and FP59 were generated and purified as previously described [52]-[54]. The LF used here is a recombinant protein having a non-native N-terminal sequence of HMAGG [55].

### Cell culture

The human HNSCC cell lines Cal27, Hep2, HN6, HN12, HN30 [56]–[58], the cervical carcinoma cell line HeLa [59], and the human colon carcinoma cell line HT29 [60] have been described. All cell lines were cultured in a humidified 5% CO_2_ environment at 37°C. Cells were maintained in Dulbecco's Modified Eagle Medium (DMEM) supplemented with 10% fetal bovine serum and 1% penicillin/streptomycin (Invitrogen, Carlsbad, CA).

### In vitro cytotoxicity assays

The cytotoxicity of PA-U2-R200A and PA-L1-I210A, with LF or FP59, was assessed using a colorimetric 3-(4,5-dimethylthiazol-2-yl)-2,5-diphenyltetrazolium bromide (MTT) assay in 96-well plates [61]. Cells exhibiting ∼40% confluence were incubated with serial dilutions of PA (0–10 nM) or PA-U2-R200A + PA-L1-I210A (0–10 nM) and either FP59 (1.9 nM) or LF (5.5 nM) to a final volume of 200 µl per well. Cell viability was determined after 48 h.

### Animals

Female Hsd:Athymic Nude-Foxn1nu mice (Harlan Laboratories Inc., Indianapolis, IN) between 4 and 6 weeks of age were used in this study. Animals were housed in a pathogen-free environment certified by the Association for Assessment and Accreditation of Laboratory Animal Care International.

### In vivo tumor xenograft model

Cells (9×10^5^ per mouse) were injected intradermally in the mid-scapular dorsal region of each mouse as described [62]. When tumors reached a volume of 50–100 mm^3^, the mice were divided into two groups of ten mice with equivalent median tumor sizes. Treatment was initiated on day 0. Mice received intraperitoneal (I.P.) injections of either 100 or 500 µl PBS or PA-U2-R200A + PA-L1-I210A + LF in 100 or 500 µl PBS, respectively. Mice bearing HN12 xenografts were administered 25 µg PA-U2-R200A +25 µg PA-L1-I210A +17 µg LF, while mice bearing Cal27, HN6 and Hep2 xenografts were treated with 20 µg PA-U2-R200A +20 µg PA-L1-I210A +13 µg LF. Mice received five injections total using a Monday, Wednesday, Friday dosing schedule. An investigator unaware of the treatment group measured the longest and shortest tumor diameters with digital calipers (FV Fowler Company, Inc., Newton, MA). Tumor volume was estimated using the equation V  =  (length in mm * (width in mm)^2^)/2 [63]. The statistical significance of differences in tumor sizes and mouse weight was determined by the two-tailed Student's t-test using Microsoft Excel software. During the 11-day treatment period, no animals in either the control or treatment groups met the criteria for early euthanasia: frank tumor ulceration or tumor mass exceeding 10% of body weight.

### Histopathological analysis

HN12 (n = 5) and Hep2 (n = 4) tumor-bearing nude mice were treated I.P. with 100 or 500 µl PBS or with 15 µg PA-U2-R200A +15 µg PA-L1-I210A +10 µg LF in 100 or 500 µl PBS on days 0, 2 and 4. The mice were euthanized by CO_2_ inhalation 24 h after the last injection. Tumors were excised, fixed in 4% paraformaldehyde for 24 h, embedded in paraffin, sectioned, and stained with hematoxylin and eosin (H&E). Sections with identifiable carcinoma cells in the H&E sections were also stained with a monoclonal rabbit anti-mouse PECAM-1 (Santa Cruz Biotechnology, Inc., Santa Cruz, CA) and a polyclonal rabbit anti-human Ki67 (Novocastra Laboratories, Ltd., Newcastle, UK). TUNEL staining was performed by Histoserv, Inc. (Germantown, MD). Images were captured using an Aperio T3 Scanscope (Aperio Technologies, Vista, CA) and were quantified using Aperio Imagescope Software (Aperio Technologies, Vista, CA) by a blinded investigator. Statistical significance of differences for apoptosis, cellular proliferation and tumor vascularization were determined using the Student's t-test, two-tailed, using GraphPad Prism software. Statistical significance of differences for necrosis was determined using the Mann-Whitney U-test using GraphPad Prism software.

## Results

**Figure 1:**
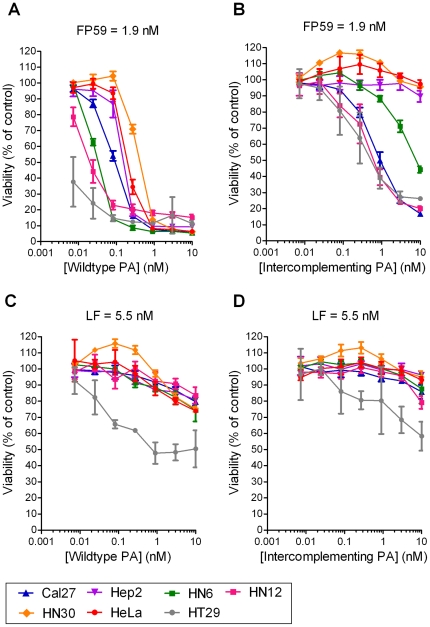
Cytotoxicity of intercomplementing anthrax lethal toxins to human HNSCC cell lines. Cal27 (blue triangles), Hep2 (purple triangles), HN6 (green squares), HN12 (purple squares), and HN30 (yellow diamonds) cells were incubated with increasing concentrations of wildtype PA in combination with FP59 (**A**), intercomplementing PA (PA-U2-R200A + PA-L1-I210A) in the presence of FP59 (**B**), wildtype PA with LF (**C**) or intercomplementing PA with LF (**D**) for 48 h. The cell viability was then measured using an MTT assay. HT29 colon carcinoma (grey circles) and HeLa (red circles) cells were used as a positive and negative controls, respectively [52], [74]. Cell survival is expressed as mean viability ± standard deviation of the mean.

We first explored if HNSCC cells express functional anthrax toxin receptors by exposing the five human HNSCC cell lines, Cal27, Hep2, HN6, HN12, and HN30 to increasing concentrations of PA in combination with 1.9 nM FP59 (Figure 1A). FP59 is a fusion protein consisting of LF residues 1–254 with the ADP-ribosylation domain of *Pseudomonas* exotoxin A. When translocated to the cytoplasm via PA, FP59 efficiently kills all cells by ADP-ribosylation and inhibition of translation elongation factor 2 [64]. PA in combination with FP59 killed all HNSCC cell lines with an LD_50_ ranging from less than 7 to 400 pM demonstrating the presence of functional anthrax toxin receptors. To assess if these HNSCC cell lines also express both functional uPA and MMP cell surface proteolytic activity, we next exposed the five HNSCC cell lines to the same concentration of FP59 in combination with increasing concentrations of intercomplementing PA (PA-U2-R200A + PA-L1-I210A) (Figure 1B). Three of the HNSCC cell lines (Cal27, HN6, HN12) were sensitive to the intercomplementing toxin (LD_50_, 0.5 nM to 8 nM), demonstrating functional uPA and MMP expression, while the two other cell lines, Hep2 and HN30 were resistant, indicating the absence of either functional uPA or MMP activity, or both. We next determined if the five HNSCC cell lines were dependent on a functional MEK/MAPK pathway for growth in culture by incubating them with 5.5 nM LF in combination with increasing concentrations of either wildtype PA (Figure 1C) or intercomplementing PA (Figure 1D). None of the HNSCC cell lines were sensitive to the two toxin combinations, showing that MEK activity is dispensable for HNSCC cell growth in culture.

**Figure 2:**
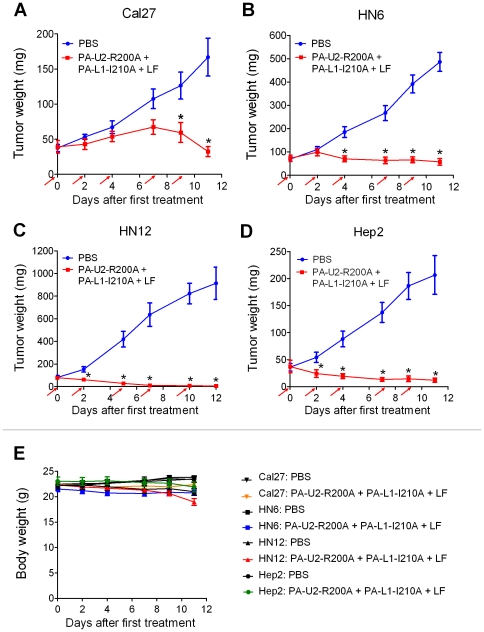
Tumoricidal activity of intermolecular complementing PA to human HNSCC. Nude mice bearing intradermal Cal27 (**A**), HN6 (**B**), HN12 (**C**), and Hep2 (**D)** HNSCC xenografts were injected intraperitoneally with either PBS (blue lines) or PA-U2-R200A + PA-L1-I210A + LF (red lines) at the time points indicated by the red arrows. (**E**) Body weight change over time in all groups. The data are expressed as mean tumor weight ± standard error of the mean; *, P<0.01. Ten mice were used per tumor and treatment group.

Cal27, Hep2, HN6, and HN12 HNSCC cell lines form solid tumors when xenografted to immunocompromized mice and were therefore suitable for assessing the efficacy of the intercomplementing toxin for HNSCC treatment *in vivo*. The four cell lines were transplanted intradermally to nude mice, and solid tumor nodules constituting 0.25 to 0.5 percent of the total body weight were allowed to form. The mice thereafter were treated three times per week with intraperitoneal injections of either intercomplementing toxin in combination with LF or with PBS as a control (Figure 2). Cal27, HN6, and HN12 tumors were all efficiently treated with the intercomplementing toxin, consistent with their expression of functional cell surface uPA and MMP activities in cell culture (Figure 2A-C). Average tumor sizes in toxin-treated mice ranged from 0.6 to 26 percent of the average tumor sizes of PBS-injected mice at treatment cessation. Interestingly, although Hep2 cells did not express uPA or MMP activity sufficient for intercomplementing toxin activation in culture (see above), the Hep2 tumors were treated efficiently *in vivo*, with the average tumor size of toxin-treated mice being just six percent of the average tumor sizes of PBS-injected mice at treatment cessation (Figure 2D). The greatest response to the intercomplementing toxin was observed with HN12-bearing mice, with 40 percent of the mice remaining tumor-free when observed for up to one year after treatment cessation.

The toxin was generally well tolerated. Two of 40 (five percent) of the toxin-treated mice died within the treatment period, whereas no death was observed in PBS-treated groups (P = N. S., Chi-square test, two tailed). The largest weight loss in toxin-treated mice as compared to PBS-treated mice (Figure 2E) was observed at day 11 in all trials. Excluding the mass attributed to tumor burden, the average body weight of all toxin treated mice was 8.3 percent lower than the average body weight for all mice in PBS-treatment groups (P<0.0004, Student's t-test, two-tailed).

**Figure 3:**
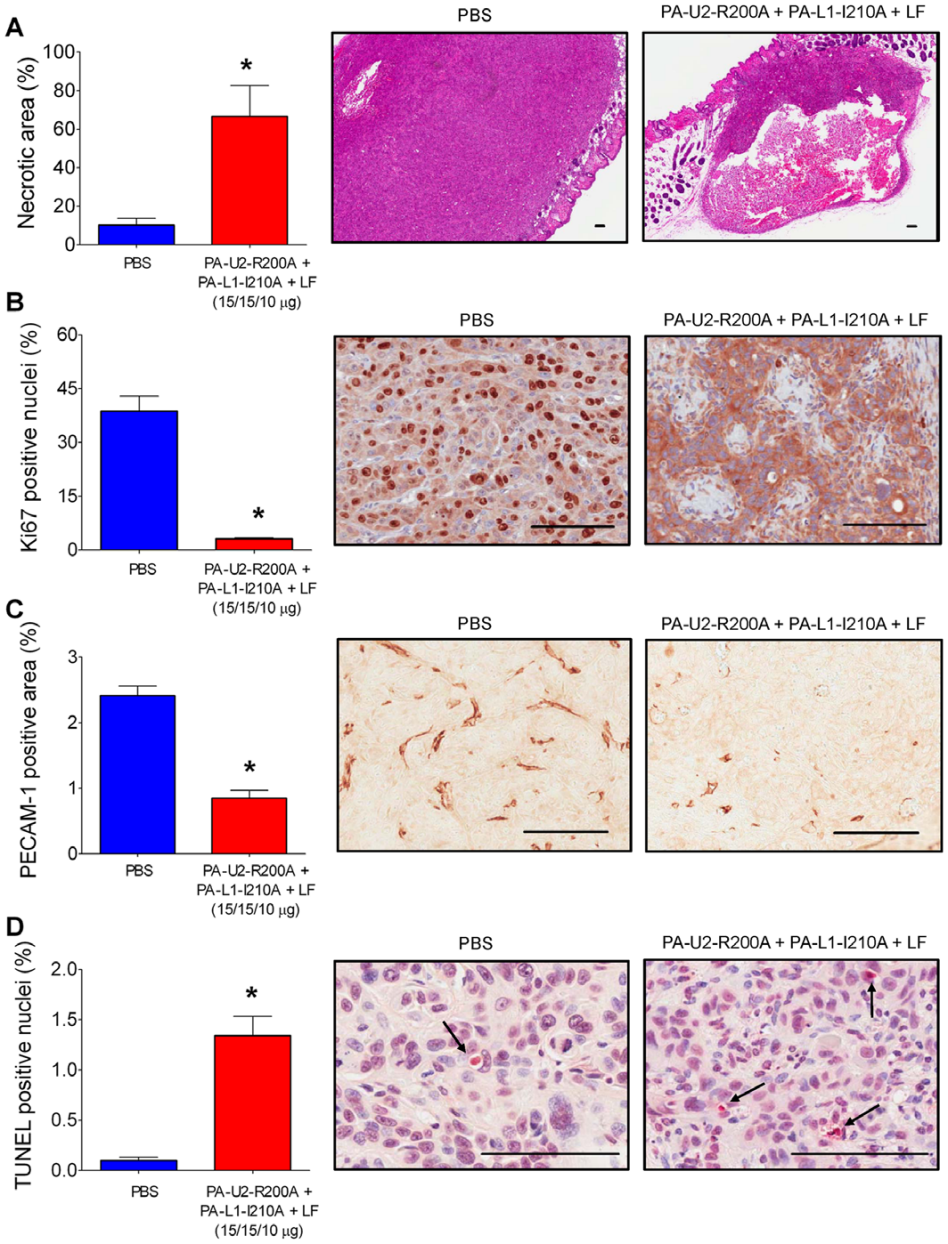
Increased necrosis and apoptosis and decreased proliferation and vessel density of human HN12 xenografts in intercomplementing toxin-treated mice. Necrosis (**A**), proliferation (**B**), tumor vascularization (**C**), and apoptosis (**D**) of HN12 xenografts 5 days after initiation of systemic treatment with either PBS (blue bars and left panels) or intercomplementing toxin (red bars and right panels). **A**, hematoxylin and eosin staining. **B**, Ki67 staining. **C**, PECAM-1 staining. **D**, TUNEL staining. The arrows in D highlight examples of TUNEL positive cells. Columns, mean; bars, standard error of the mean, *, P<0.05. In all cases, representative images are shown. Scale Bars; 100 µM.

**Figure 4:**
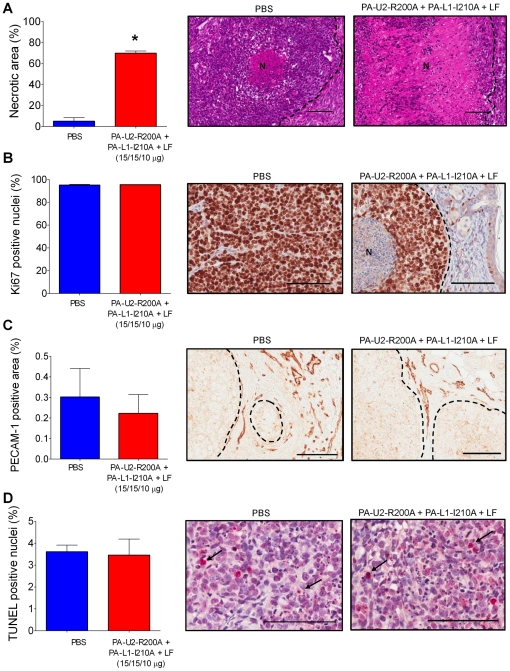
Intercomplementing toxin induces massive tumor necrosis in xenografted human Hep2 tumors, but does not affect proliferation, vascularization or apoptosis in viable tumor areas. Necrosis (**A**), proliferation (**B**), tumor vascularization (**C**), and apoptosis (**D**) of Hep2 xenografts five days after initiation of systemic treatment with either PBS (blue bars and left panels) or intercomplementing toxin (red bars and right panels). **A**, hematoxylin and eosin staining. **B**, Ki67 staining. **C**, PECAM-1 staining. **D**, TUNEL staining. N in **A** and **B** indicates necrotic area. Tumor margins in **A**–**C** are indicated with dotted lines. Arrows in **D** highlight examples of TUNEL positive cells. Columns, mean; bars, standard error of the mean, *, P<0.05. In all cases, representative images are shown. Scale Bars; 100 µM.

We next examined the mechanistic basis for the potent systemic anti-HNSCC activity of the intercomplementing toxin. For this purpose HN12 (sensitive to intercomplementing toxin administered with FP59 *in vitro*) and Hep2 cells (insensitive to intercomplementing toxin administered with FP59 *in vitro*) were transplanted to mice, and established tumors were treated three times with toxin or PBS. The tumors were then excised (at day 5, as per Figure 2), and histological sections were generated, scanned, and subjected to an unbiased quantitative histomorphometric analysis. No tumor tissue was identified in histological sections from two of five examined HN12 toxin-treated tumors, indicating complete tumor regression. The three remaining toxin-treated HN12 tumors presented with a four-fold increase in the area of tumor necrosis, as compared to untreated tumors (Figure 3A). Cell proliferation in the remaining viable areas of these toxin-treated tumors was more than twelve-fold reduced, as determined by staining of the cell proliferation marker, Ki67 (Figure 3B). Vessel density was likewise reduced by three-fold (Figure 3C), while the apoptotic index was increased 13-fold (Figure 3D). Interestingly, unlike the case for the HN12 tumors examined above, the intercomplementing toxin primarily targeted Hep2 tumors by inducing necrosis (Figure 4). Thus, necrosis was increased by more than 13-fold (Figure 4A), while cell proliferation, vessel densities, and apoptotic indices in the remaining viable areas of the xenografted Hep2 tumors were all unaffected by treatment with intercomplementing toxin (Figure 4B-D). The retention of these latter tumor properties was notable given the striking decrease in tumor mass (Figure 2D, day 5).

## Discussion

Research over the last decade has led to the generation of several modified anthrax toxin-based candidate anti-tumor compounds that exploit the signature overexpression of extracellular proteases by tumor cells and the cellular components of the tumor stroma to achieve tumor selectivity [52], [53], [65]–[74]. In the current study we performed a comprehensive analysis of the suitability of one of these compounds: an intercomplementing toxin for the treatment of HNSCC. A unique property of this toxin is its inclusion of no less than three specificity determinants: the requirement for both cell surface uPA activity and cell surface MMP activity, combined with selective toxicity to cells dependent on the MEK/MAPK kinase pathway for survival [42], [52].

Xenograft studies revealed that the intercomplementing toxin displayed excellent systemic antitumor activity towards all of the four analyzed HNSCC cell lines. By using a treatment regimen consisting of five intraperitoneal toxin injections, we achieved effects ranging from tumor stasis to complete tumor eradication, as defined by the absence of relapse in mice that were followed for up to one year after treatment cessation.

A notable observation in the current study was that sensitivity to LF or expression of functional cell surface uPA and MMP activity by cultured HNSCC cell lines were poor predictors of the *in vivo* sensitivity of the tumor to the intercomplementing toxin. Thus, although all of the four tumors resulting from the HNSCC cell lines were efficiently treated with the intercomplementing toxin when xenografted to mice, none of the cell lines displayed significant sensitivity to LF in culture (wildtype PA with LF, Figure 1C), and one cell line did not express sufficient cell surface uPA and MMP activity for functional PA heptamer formation (Hep2 cells, intercomplementing toxin with FP59, Figure 1B). Two possible, and not mutually exclusive, explanations for this observation can be offered. First, the repertoire of cell surface proteases expressed by the tumor cells or their requirement for the ERK/MAPK pathway for proliferation and survival may differ in culture and *in vivo*. Secondly, the intercomplementing toxin may exert its anti-tumor activity by targeting the cellular component of the HNSCC tumor stroma (tumor-associated inflammatory cells, fibroblasts, endothelial cells) [75]. In direct support of the latter, we have previously shown that LF can efficiently impair the growth of a xenografted immortalized ovarian cell line that was made genetically deficient in anthrax toxin receptors [69]. It also remains a possibility that activation of the intercomplementing toxin by proteases different from uPA and MMPs account for the *in vivo* efficacy of the intercomplementing toxin. This, however, is unlikely to be the case. First, cleavage of MMP-activated PA and subsequent cellular intoxication can be blocked completely by the synthetic MMP inhibitors BB94 (Batimastat), BB2516 (Marimastat), and GM6001, as well as tissue inhibitor of matrix metalloproteinases-2. Cleavage of the uPA-activated PA and cellular intoxication on the other hand can be prevented by plasminogen activator inhibitor-1 and by antibodies and also by protein fragments that block the binding of uPA to its cellular receptor. Finally, using mice genetically deficient in components of the plasminogen activation system, we have previously shown that the anti-tumor activity of the uPA-activated PA *in vivo* is dependent on cell surface uPA activity [52], [53], [73], [74], [76].

Histological analysis of HN12 and Hep2 xenografts revealed markedly different effects of systemic intercomplementing toxin treatment on the two tumors. While toxin-treated HN12 xenografts displayed decreased proliferation and an increase in both apoptotic and necrotic cell death, neither proliferation or apoptosis was affected by toxin treatment of Hep2 xenografts. Rather, the potent anti-tumor activity of the intercomplementing toxin was caused exclusively by the induction of tumor necrosis. In light of the inability of cultured Hep2 cells to express sufficient uPA and MMP activity for cellular intoxication, it is tempting to speculate that tumor necrosis in Hep2 xenografts is induced by a vascular collapse caused by direct targeting of the tumor vasculature or other essential cellular components of the tumor stroma.

The anti-tumor efficacy of the intercomplementing toxin and tolerability at administered doses highlight the therapeutic potential of this agent. Procedures for recombinant expression and purification of large quantities of anthrax toxins in avirulent strains of *Bacillus anthracis* and *Eschericia coli* are already established and will not represent an impediment to the therapeutic development of the intercomplementing toxin for treatment of HNSCC. The pharmacology and pharmacokinetics of wildtype PA and LF are well studied, and the intercomplementing PA could be expected to display pharmacological and pharmacokinetic properties similar to or even more favorable that those of wildtype PA due to enhanced plasma stability [69], [77]–[79]. Systematic animal toxicity studies required for the clinical introduction of the intercomplementing toxin are in progress (D. E. P., S. H. L., and T. H. B., unpublished data).

The absence of correlation between *in vivo* efficacy and sensitivity of cultured HNSCC cell lines to the intercomplementing toxin discussed above raise interesting questions as to the potential for prescreening HNSCC patients for treatment. Based on our findings, assaying toxin-sensitivity of cultured primary tumor cell explants may be of limited value. However, determination of tumor cell and stromal cell uPA and MMP protein expression or enzymatic activity in resected tumors or needle biopsies may be a clinically useful predictor of treatment efficacy.

In summary, we have shown that an intercomplementing toxin specifically targeting ERK/MAPK-dependent cells with high cell surface uPA and MMP activity holds promise as a novel candidate drug for the treatment of HNSCC. Future research towards the clinical development of this toxin is warranted.
